# Molecular Mechanisms of Wound Healing: The Role of Medicinal Plants

**DOI:** 10.3390/life15111748

**Published:** 2025-11-14

**Authors:** Merlin Esad, Ivica Dimov, Mariya Choneva, Mihaela Popova, Vesela Kokova, Elisaveta Apostolova, Anelia Bivolarska

**Affiliations:** 1 Department of Medical Biochemistry, Faculty of Pharmacy, Medical University of Plovdiv, 4002 Plovdiv, Bulgaria; ivica.dimov@mu-plovdiv.bg (I.D.); mariya.choneva@mu-plovdiv.bg (M.C.); anelia.bivolarska@mu-plovdiv.bg (A.B.); 2 Independent Researcher, 37081 Goettingen, Germany; 3 Department of Pharmacology, Toxicology and Pharmacotherapy, Faculty of Pharmacy, Medical University of Plovdiv, 4002 Plovdiv, Bulgaria; vesela.kokova@mu-plovdiv.bg (V.K.);; 4 Research Institute, Medical University of Plovdiv, 4002 Plovdiv, Bulgaria

**Keywords:** wound healing, molecular mechanisms, phytotherapy, medicinal plants

## Abstract

Wound healing is a tightly regulated biological process involving hemostasis, inflammation, proliferation, and tissue remodeling. When these phases are disrupted, wound repair can be delayed or become chronic. Key signaling pathways, including NF-κB, JAK/STAT, and MAPK, coordinate immune activation, cytokine expression, cell proliferation, and tissue repair. Medicinal plants and their bioactive compounds, such as flavonoids, alkaloids, tannins, and other phytoconstituents, have demonstrated significant anti-inflammatory, antioxidant, and immunomodulatory effects that modulate these pathways. Tannins contribute to repair through neutralization of reactive oxygen species (ROS), activation of antioxidant enzymes, and metal-chelating activity. Alkaloids, including tetrandrine, oxymatrine, and berberine, inhibit NF-κB signaling, thereby reducing pro-inflammatory cytokines such as IL-1β and TNF-α. Flavonoids regulate inflammatory mediators and enzymes, including COX and phospholipase A2, while also protecting against oxidative stress and stimulating fibroblast and keratinocyte proliferation—key steps in tissue regeneration. Collectively, these compounds accelerate wound closure by reducing oxidative stress and promoting cellular proliferation and migration. Thus, medicinal plants represent promising complementary approaches to wound management. Future research should focus on developing advanced drug delivery systems to enhance the stability, bioavailability, and targeted action of plant-derived compounds. Localized and biomaterial-based strategies show promise for sustained release at the wound site, and further preclinical and clinical studies are required to ensure their safety, reproducibility, and efficacy.

## 1. Introduction

The skin is the largest organ in the human body by surface area. It protects internal tissues and organs from mechanical damage, microbial infection, ultraviolet radiation, and extreme temperatures. This makes it highly susceptible to injury with significant impacts on both individual patients and healthcare economics [1]. Maintaining skin homeostasis is important for maintaining the stability of the entire internal environment. The skin barrier is the first line of defense of the human body, and its main function is to prevent water loss and the entry of harmful external substances into the body. Only when skin homeostasis is well maintained can the skin barrier function properly and protect the body from external threats [2]. The epidermis is the outer, impermeable layer that contains sebaceous glands, sweat glands, and hair follicles. The dermis is rich in extracellular matrix (ECM), capillary network, and mechanoreceptors and provides the skin with strength, nutrients, and immune protection. Subcutaneous adipose tissue underlies the dermis and functions as an energy reserve. It is also a constant source of growth factors for the dermis [1].

A wound is an injury characterized by the loss or disruption of the normal continuity of the skin or mucosal tissue, resulting from physical, thermal, or surgical causes [3]. Wounds are among the most common diseases affecting the skin. They form after tissue injury, usually by external agents, causing severe damage to the epidermis and underlying connective tissue [4]. Skin repair requires a complex synchronization of several different cell types in sequential steps.

Wounds can be classified in various ways based on their etiology, localization, type of injury, functional changes, depth, tissue loss, or appearance. Broadly, wounds are divided into two categories: acute and chronic. Acute wounds occur suddenly as a result of trauma, surgery, or burns. They are characterized by rupture of blood capillaries and the activation of hemostasis (e.g., cuts, abrasions, burns). Chronic wounds are usually associated with underlying diseases and are defined by deviations from the physiological healing process [5].

**Figure 1:**
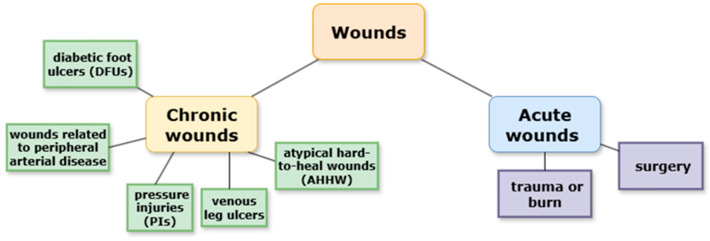
Classifications of wounds.

In recent years, chronic wounds have emerged as a global health concern. Chronic or hard-to-heal wounds are commonly defined as wounds that fail to reduce in size by more than 40–50% or fail to heal within one month. The global prevalence of chronic wounds is estimated at 1.51 to 2.21 per 1000 individuals, and the incidence is expected to rise with the aging global population. Chronic wounds may have diverse etiologies but are generally classified into the following categories: diabetic foot ulcers (DFUs), wounds associated with peripheral arterial disease, venous leg ulcers, pressure injuries (PIs), and atypical hard-to-heal wounds (AHHW) (Figure 1). Even after apparent healing, recurrence rates remain high, with up to 40% of DFUs and 69% of venous leg ulcers relapsing within one year [6]. Recent studies have reported that chronic wounds can undergo malignant transformation, with cases of long-standing ulcers progressing to squamous cell carcinoma, highlighting the need for early diagnosis and management. Therefore, careful monitoring of non-healing wounds is essential to prevent severe outcomes [7].

## 2. Materials and Methods

This review was conducted as a narrative literature review with an emphasis on identifying experimental and clinical studies investigating medicinal plants in wound healing. The aim of this review is to summarize the mechanisms, bioactive compounds, and therapeutic potential of medicinal plants in wound healing. The review is organized into sections covering the pathophysiology of wound healing, the signaling pathways involved, the phytochemicals that contribute to tissue repair, and the pharmacological activities of selected medicinal plants. In addition, the review outlines the methods commonly used to study the molecular and cellular mechanisms underlying the wound-healing process. The literature search was conducted using the following electronic databases: PubMed (https://pubmed.ncbi.nlm.nih.gov, accessed on 10 June 2025), Scopus (https://www.scopus.com/pages/home?display=basic&zone=header&origin=sbrowse#basic, accessed on 10 June 2025), and Google Scholar (https://scholar.google.com, accessed on 10 June 2025). A comprehensive search was carried out to identify publications appearing between 2000 and 2025 using the keywords “wound healing,” “phytotherapy”, and “medicinal plants”. From the initial 120 records identified, duplicates were excluded, leaving 100 articles for title and abstract screening. After reviewing 10 full texts, 6 studies were retained for inclusion. Studies were most often excluded due to insufficient primary evidence or non-wound-related outcomes. Only peer-reviewed articles in English were included, while conference abstracts and non-English articles were excluded. Data were synthesized narratively and organized thematically to highlight key findings.

## 3. Discussion

### 3.1. Stages of Wound Healing

**Figure 2:**
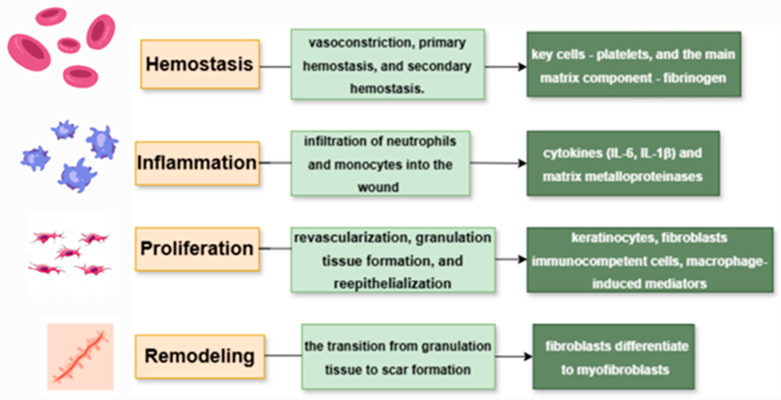
Phases of wound healing and the main cell components involved in them.

Wound healing, one of the most complex processes in the human body, proceeds through four overlapping phases: hemostasis, inflammation, proliferation, and remodeling (Figure 2).

#### 3.1.1. Hemostasis

Hemostasis is the first stage of wound healing, responsible for stopping bleeding after vascular injury. It occurs in three sequential steps: vasoconstriction, primary hemostasis, and secondary hemostasis. The main cells involved are platelets, while the key extracellular matrix component is fibrinogen. In their inactive state, platelets neither adhere to the vascular wall nor aggregate with each other. When there is a vascular injury or inflammation, it results in a coordinated series of events, including platelet adhesion, aggregation, and promotion of coagulation [8].

Fibrinogen (coagulation factor I) is produced by the liver and circulates in the bloodstream. Upon vascular injury, the coagulation cascade is activated, leading to the conversion of fibrinogen into fibrin, a reaction catalyzed by the serine protease thrombin (factor IIa). It is also present in platelets but does not degrade into fibrin fibers until activated; fibrin fibers are an essential structural component of the blood clot [1].

Following injury, blood vessels rapidly constrict to minimize bleeding. This is achieved through reflex contraction of vascular smooth muscle and the release of vasoconstrictors such as endothelin from damaged endothelial cells. Additionally, circulating catecholamines—epinephrine, norepinephrine, serotonin, thromboxane A_2_, and other prostaglandins released by injured cells—further regulate vasoconstriction [9].

Primary hemostasis involves platelet aggregation and the formation of a platelet plug in response to exposed subendothelial collagen. Secondary hemostasis corresponds to the coagulation cascade, in which soluble fibrinogen is converted into an insoluble fibrin mesh. The platelet plug, stabilized by the fibrin network, forms a thrombus that halts bleeding. This thrombus also serves as both a source of growth factors and a temporary scaffold that supports the migration of cells involved in wound repair [1].

#### 3.1.2. Inflammation

The inflammatory phase overlaps with the initial stage of hemostasis and occurs within the first 72 h after tissue injury [10]. This phase is primarily characterized by a complex cascade of molecular signals that facilitate the infiltration of neutrophils and monocytes into the wound in order to prevent further tissue damage and to eliminate pathogenic organisms and cellular debris [10,11,12].

The inflammatory phase can be divided into two distinct stages: early inflammation and late inflammation. During early inflammation, clearance of apoptotic bodies and cellular debris is mediated by macrophages and supported by neutrophils as part of the inflammatory response [13]. Cytokines such as interleukin IL-6 and IL-1β, as well as matrix metalloproteinases (MMPs), are released [14]. Deep dermal fibroblasts produce significantly higher levels of IL-6 compared to superficial fibroblasts, which contributes to differences in healing between superficial and deep wounds. IL-6 signaling promotes interactions between profibrotic fibroblasts and keratinocytes. In addition, it stimulates macrophages and monocytes to produce pro-inflammatory cytokines through activation of the mitogen-activated protein kinase (MAPK) and nuclear factor kappa B (NF-κB) pathways MAPK and NFκB pathways. Monocytes and macrophages secrete IL-1β and tumor necrosis factor-alpha (TNF-α) in response to IL-6, which induces fibroblasts to produce keratinocyte growth factor (KGF) [15]. KGF is a potent activator of keratinocytes, enhancing their proliferation and migration [16]. Subsequently, keratinocytes produce oncostatin M (another member of the IL-6 cytokine family), which acts in a paracrine manner to stimulate profibrotic signal transducer and activator of transcription 3 (STAT3) signaling in dermal fibroblasts [17]. The late inflammatory phase involves anti-inflammatory M2 macrophages, which release complement components and several growth factors, including vascular endothelial growth factor (VEGF), insulin-like growth factor-1 (IGF-1), fibroblast growth factor (FGF), epidermal growth factor (EGF), and transforming growth factor-β (TGF-β), along with anti-inflammatory cytokines such as IL-10 [1,18]. The final immune cells to arrive at the wound site are lymphocytes. They play a crucial role in regulating collagenase activity, which is essential for collagen remodeling, extracellular matrix (ECM) synthesis, and its degradation [18].

#### 3.1.3. Proliferation

Following the resolution of the inflammatory response, the proliferative phase begins. This stage is characterized by revascularization, granulation tissue formation, and re-epithelialization of the wound surface. During physiological healing, keratinocytes located at the wound edges initiate a centripetal migratory process within hours after tissue injury. Simultaneously, epithelial stem cells residing in the basal layer of the epidermis and in the outer root sheath of hair follicles begin active proliferation approximately 2–3 days post-injury [19].

Angiogenesis and re-epithelialization are modulated by a wide range of chemical and physical mediators, many of which are secreted by immunocompetent cells, particularly anti-inflammatory macrophages. Restoration of the vascular network represents a critical aspect of the proliferative phase of tissue regeneration. Neoangiogenesis-the process of forming new blood vessels-occurs through sequential induction of capillary sprouting followed by vascular anastomosis [20]. Anti-inflammatory macrophages exert a central regulatory role, not only by secreting VEGF, which induces endothelial proliferation, but also by expressing specific transmembrane proteins that have been shown to participate in vascular anastomosis. This macrophage-driven angiogenic activity is essential for effective tissue regeneration [12,21].

Granulation tissue formation, composed primarily of type III collagen, fibroblasts, and newly formed blood vessels, occurs in parallel with angiogenesis. Fibroblasts are the main cells responsible for synthesizing granulation tissue. They are activated by a variety of macrophage-derived mediators, including platelet-derived growth factor β (PDGF-β), TNF-α, and interleukins IL-1 and IL-6, which stimulate the expression of pro-re-epithelialization factors in fibroblasts [12]. In the absence of IL-6, the inflammatory response is insufficient, while angiogenesis, collagen deposition, and re-epithelialization are significantly impaired. Additionally, fibroblasts are highly responsive to (TGF-β), a key mediator produced in large amounts by early regulatory, pro-regenerative macrophages [22]. Re-epithelialization is modulated by both fibroblasts within the granulation tissue and pro-regenerative macrophages. This process is initiated by the action of EGF, keratinocyte growth factor (KGF), and transforming growth factor α (TGF-α), produced by platelets, keratinocytes, and activated anti-inflammatory macrophages [12].

#### 3.1.4. Remodeling

The remodeling phase begins several weeks after injury and may continue for up to one year. This stage marks the transition from granulation tissue to scar formation. The main features of this phase are the reduction of angiogenesis and the replacement of type III collagen in the granulation tissue with the stronger type I collagen [12]. The remodeling phase is primarily driven by myofibroblasts, which differentiate from fibroblasts in response to mechanical tension and TGF-β signaling. These cells are responsible for wound contraction [23].

## 4. Inflammatory Mediators, Receptors, and Key Signaling Pathways

### 4.1. Cytokines

**Figure 3:**
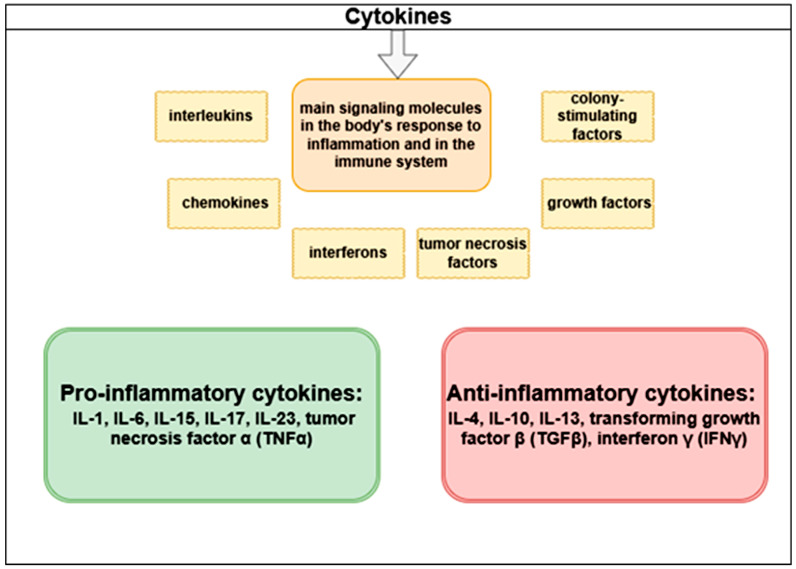
Classification of cytokines.

Cytokines are the principal signaling molecules involved in the body’s response to inflammation and in the regulation of the immune system. They are classified into interleukins, chemokines, interferons, tumor necrosis factors, growth factors, and colony-stimulating factors. The classification of cytokines is presented in Figure 3.

TNFα is one of the major pro-inflammatory cytokine mediators, exerting multiple effects on different cell types. Activated macrophages and T-cells, in response to various inflammatory stimuli, secrete TNFα. Uncontrolled or chronic secretion of TNFα can contribute to the development of several diseases, including chronic inflammatory disorders, cancer, and autoimmune diseases [24 ]. TNF-α mediates its biological effects primarily through two receptor types: TNFR1 and TNFR2. TNFR1 is broadly expressed across nearly all tissues and represents the principal signaling receptor for TNF-α, while TNFR2 shows a more restricted expression pattern, being mainly present in immune cells, where it contributes to more limited functional responses [25].

Interleukins represent a major class of cytokines that play a key role in immune modulation. IL-6 is a critical mediator of acute inflammatory responses, and its uncontrolled production has been associated with numerous chronic inflammatory conditions [26]. The primary cellular sources of IL-6 during inflammation are monocytes, macrophages, and T-lymphocytes. Its production and secretion are regulated by several transcription factors, particularly NF-κB and activator protein-1 (AP-1) [27,28]. When IL-6 binds to its membrane-bound receptor (IL-6R), it triggers dimerization of gp130 and activates associated Janus kinases (JAKs). These kinases phosphorylate gp130, thereby facilitating the recruitment and activation of transcription factors STAT3 and STAT1, as well as other signaling molecules, including phosphatidylinositol-3-kinase (PI3K), Ras-MAPK, and Src-homology-2-containing protein tyrosine phosphatase 2 (SHP2) [27].

**Figure 4:**
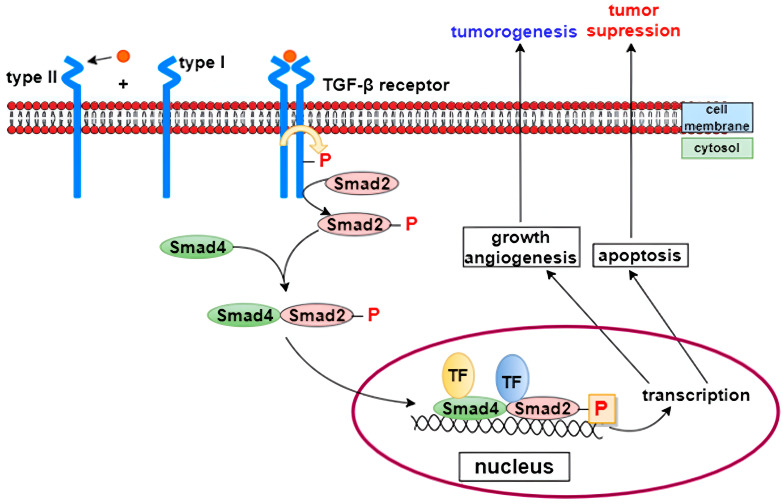
TGF-β signaling pathway [31]. TGF-β is a key regulatory cytokine involved in controlling inflammation and tissue repair. Activated TGF-β dimers bind to type II receptors, which phosphorylate and activate type I receptors. The activated receptor complex then phosphorylates Smad transcription factors, which associate with coactivators or repressors to regulate the expression of numerous target genes involved in inflammation, fibroblast activation, and extracellular matrix synthesis.

TGF-β is a regulatory cytokine that plays a crucial role in controlling inflammation [29]. Alterations in its activity can lead to chronic inflammation [27]. Activated TGF-β dimers signal through their receptors, type I and type II, which are serine/threonine kinase receptors. Upon ligand binding, the type II receptor phosphorylates the type I receptor, which subsequently phosphorylates Smad transcription factors. Activated Smad complexes recruit transcriptional coactivators and repressors, enabling the simultaneous activation or suppression of hundreds of target genes [30] (Figure 4).

### 4.2. Chemokines

Chemokines, the chemoattractant cytokines, act in a coordinated manner and participate in numerous biological processes such as cell invasion, motility, survival, and interactions with the extracellular matrix during immunological and inflammatory responses. Chemokine receptors are G protein–coupled receptors [28].

## 5. Inflammation Signaling Pathways

### 5.1. The NF-κB Pathway

**Figure 5:**
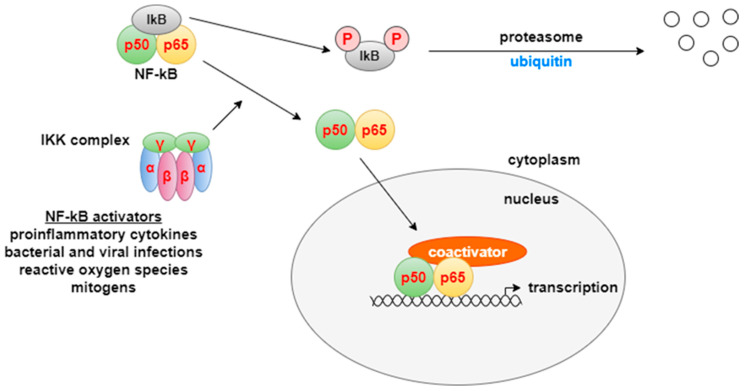
NF-κB pathway [31]. NF-κB is a transcription factor that plays a central role in regulating genes involved in inflammation, cell proliferation, differentiation, and immune responses. Under resting conditions, NF-κB (a p50/p65 heterodimer) remains inactive in the cytoplasm through binding to the inhibitory protein IκB. In response to extracellular stimuli, the IκB kinase (IKK) complex becomes activated and phosphorylates IκB on serine residues, leading to its proteasomal degradation. The released NF-κB translocates into the nucleus, where it binds to specific promoter regions and initiates the transcription of genes mediating inflammatory and immune responses.

There are two different pathways for NF-κB activation: canonical and non-canonical, which are based on different molecular mechanisms for NF-κB activation, as well as different groups of target genes involved in cell proliferation, differentiation, and immune response [32,33]. The transcription factor NF-κB is a heterodimer made up of two subunits–p50 and p65. At rest, it is inactive and is located in the cytoplasm, where it is associated with the inhibitory protein IκB. In the presence of stimuli from the external environment, the IκB kinase complex (IKK) is activated. It has a heterohexameric organization, including two α-, β-, and γ-subunits. IKK phosphorylates IκB on two serine residues, which induces ubiquitination and degradation of the inhibitor by the proteasome. After the elimination of IκB, NF-κB is released and enters the nucleus, where it binds to the promoter regions of various genes and triggers the transcription of mediators of the inflammatory response. NF-κB activity is also modified by a number of coactivators, including CREB (cAMP response element-binding protein), which help fine-tune gene expression [31] (Figure 5). The canonical NF-κB pathway plays an important role in cell survival and proliferation, epithelial–mesenchymal transformation (EMT) of tumor cells, angiogenesis, cancer metastasis, and inflammation [33]. Epigallocatechin gallate, curcumin, and apigenin act at different levels of this pathway by suppressing its activation [34].

### 5.2. JAK/STAT Pathway

**Figure 6:**
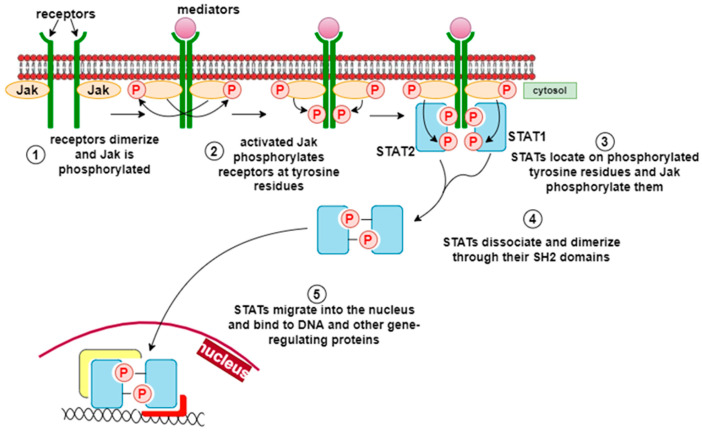
JAK/STAT pathway [31]. The JAK/STAT (Janus kinase/signal transducer and activator of transcription) pathway is activated by various cytokines, growth factors, and interferons, regulating cell proliferation, differentiation, apoptosis, and inflammatory responses. Upon ligand binding, cytokine receptors activate JAKs, which phosphorylate STAT proteins. The phosphorylated STATs form dimers that translocate to the nucleus, where they regulate the transcription of cytokine-responsive genes involved in inflammation control, innate immunity, and tissue repair.

JAK/STAT (Janus kinase/signal transducer and activator of transcription) is a signaling pathway used by multiple cytokines, growth factors, and interferons and is closely associated with cell proliferation, apoptosis, differentiation, and inflammatory responses [34]. The binding of mediators to cytokine receptors leads to cytokine-receptor interactions in the cytoplasmic domain, which induces phosphorylation of JAK and STAT proteins. Activated STAT proteins form dimers that translocate to the nucleus and modulate the expression of specific cytokine-responsive genes (Figure 6) [35]. This pathway is essential for the suppression of the inflammatory process, the initiation of innate immunity, and the coordination of adaptive immune mechanisms. Flavonoids such as curcumin, quercetin, and several types of plant polyphenolic compounds act at different levels of this pathway to suppress its abnormal activation [36].

### 5.3. MAPK Pathway

**Figure 7:**
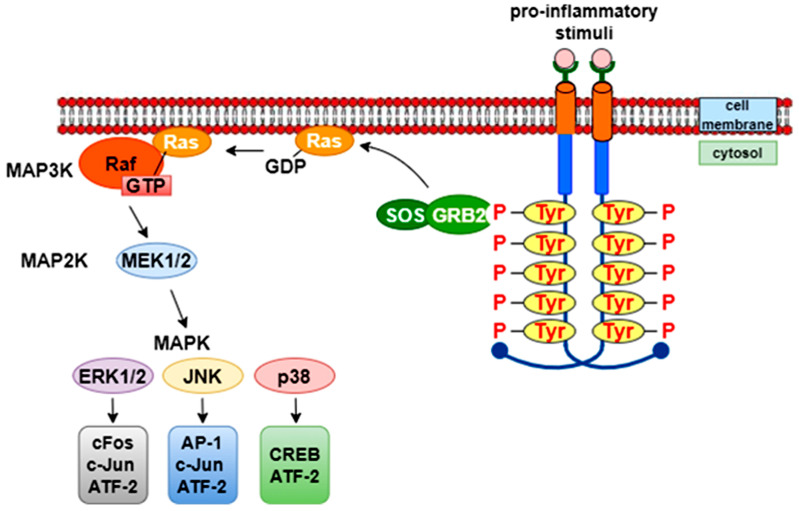
MAPK pathway.

MAPK (mitogen-activated protein kinases) are a family of serine/threonine protein kinases that include various kinases, such as ERK (extracellular signal-regulated kinase), JNK (c-Jun N-terminal kinase), and p38 MAPK. The binding of various pro-inflammatory stimuli to G-protein or tyrosine kinase receptors leads to the activation of Ras by replacing GDP with GTP, which in turn activates MAP3K (e.g., Raf). RAF kinase phosphorylates and activates the MEK enzymes (MEK1 and MEK2), which then activate MAPK (ERK, p38, or JNK) [37]. Activation of ERK stimulates the activation of transcription factors such as cFos, c-Jun, and activating transcription factor 2 (ATF-2). Similarly, activation of JNK leads to the activation of AP-1, c-Jun, and ATF-2. p38 MAPK is involved in both anti-inflammatory and pro-inflammatory processes, and its actions involve transcription factors such as CREB (cAMP response element-binding protein) and ATF-2 [38] (Figure 7). Flavonoids, such as eupatylline and phenolic compounds including vanillin, as well as some terpenoids and alkaloids, regulate this signaling pathway [39].

The mitogen-activated protein kinase (MAPK) pathway comprises a family of serine/threonine kinases, including ERK (extracellular signal-regulated kinase), JNK (c-Jun N-terminal kinase), and p38 MAPK, which regulate key cellular processes such as proliferation, differentiation, apoptosis, and inflammation. Upon stimulation by pro-inflammatory cytokines or growth factors, G-protein or tyrosine kinase receptors activate Ras, which subsequently triggers MAP3K activation. Raf phosphorylates and activates MEK1/2, which in turn activates downstream MAPKs (ERK, JNK, or p38). Activated ERK stimulates transcription factors such as c-Fos, c-Jun, and ATF-2, while JNK and p38 regulate AP-1, CREB, and other transcriptional regulators involved in stress and immune responses.

## 6. Plant-Derived Substances

Plant-derived substances belonging to a variety of chemical classes have demonstrated proven anti-inflammatory activity [40]. Modulating inflammation through the use of medicinal plants is proposed as an alternative to conventional therapeutic methods for many diseases. A number of medicinal plants have shown significant anti-inflammatory, immunomodulatory, and wound-healing properties by modulating inflammation, stimulating collagen synthesis, promoting tissue regeneration, lymphocyte activation, and apoptosis [28,41,42,43]. These include alkaloids, terpenes, phenolic compounds such as tannins, lignans, coumarins, saponins, and especially flavonoids [44] (Table 1).

### 6.1. Tannins

Within the molecular mechanisms of wound healing, certain phytochemicals exhibit antioxidant and protective effects on cells in damaged tissue. Tannins constitute a distinct class of compounds found in high concentrations in some medicinal plants used in traditional medicine. Their antioxidant and anti-inflammatory properties contribute to the acceleration of wound repair processes. Their pharmacological effects are attributed to their strong ability to bind metal ions (e.g., iron, manganese, copper), potent antioxidant activity, and capacity to interact with other biomolecules such as proteins and polysaccharides [45].

Reactive oxygen species (ROS) are byproducts of aerobic cellular metabolism. These include highly reactive superoxide anion (O_2_^−^), hydrogen peroxide (H_2_O_2_), and hydroxyl radical (·OH). “Oxidative stress” results from excessive ROS production or insufficient neutralization, potentially leading to cellular damage. Free ROS can oxidize lipids, denature proteins, and induce DNA mutations, thereby contributing to the development and progression of various diseases [46]. The mechanisms underlying the antioxidant activity of tannins include direct neutralization of ROS, activation of antioxidant enzymes, and enhancement of metal-chelating activity [47]. In vitro studies on HaCaT Cells have shown that tannin-rich extracts from *Hamamelis virginiana* possess antioxidant and anti-inflammatory activities, which may contribute to wound healing [48].

### 6.2. Alkaloids

In the context of molecular mechanisms underlying wound healing, certain phytochemicals exhibit anti-inflammatory effects by modulating signaling pathways critical for cellular responses in damaged tissue. Among these are alkaloids, known for their anti-inflammatory properties. Alkaloids are classified based on the nature of the nitrogen atom in their structure. Alkaloids containing a nitrogen atom within a heterocyclic ring are termed true alkaloids. Compounds with nitrogen atoms not incorporated into a heterocyclic ring are referred to as protoalkaloids, while compounds with or without heterocyclic rings that are not derived from amino acids are classified as pseudoalkaloids [49]. Alkaloids such as tetrandrine, oxymatrine, and berberine inhibit the NF-κB pathway in activated B cells, leading to reduced expression of pro-inflammatory cytokine genes IL-1β and TNF-α [50].

Oxymatrine improves skin inflammation symptoms by upregulating the expression of suppressor of cytokine signaling 1 and inhibiting the activation of the JAK-STAT3 pathway. Treatment with oxymatrine in mice showed significantly reduced serum levels of IgE, TNF-α, IL-4, and IL-7 [51].

### 6.3. Flavonoids

Flavonoids exhibit anti-inflammatory effects by inhibiting the production of inflammatory mediators, modulating the arachidonic acid pathway, and suppressing the activity of enzymes such as cyclooxygenase (COX), lipoxygenase (LOX), and phospholipase A2 [52]. They also possess potent antioxidant activity, neutralizing reactive oxygen species (ROS) and regulating cellular signaling pathways associated with inflammation [47,53]. Due to these properties, flavonoids can support the wound healing process in the skin by reducing local inflammation, protecting cells from oxidative stress, and promoting the proliferation and migration of keratinocytes and fibroblasts—key steps in tissue repair.

In a study conducted by Aly et al., the total phenolic (TPC) and flavonoid (TFC) contents of *G. glabra* and *S. japonica* flavonoid-rich fractions were quantified using gallic acid and quercetin equivalents, respectively, with TPC values of 71.61 ± 3.23 and 70.29 ± 1.94 μg GAE/mg, and TFC values of 46.99 ± 2.57 and 49.91 ± 2.36 μg QE/mg. The topical application of formulations containing *G. glabra* and *S. japonica* flavonoid-rich fractions, particularly the combined preparations, significantly enhanced wound contraction compared to individual extracts and the untreated control. Among the treatments, the combination demonstrated the most pronounced effect, accelerating wound closure by up to 3.8-fold on day 14, indicating a synergistic effect of the two flavonoid-rich fractions in promoting wound healing [54].

### 6.4. Terpenes

Triterpenes, belonging chemically to the class of isoprenoids, are widely distributed in plants. Studies have shown that triterpenes from medicinal plants enhance wound healing by modulating pro- and anti-inflammatory mediators, chemokines, and growth factors, as well as promoting granulation tissue formation, re-epithelialization, and wound contraction [55]. The natural triterpene lupeol is a bioactive found in various edible plants. Several studies have shown the pharmacological potential of lupeol, including antioxidant, anti-inflammatory, and wound-healing effects in experimental models in vivo and in vitro [56].

A comparative metabolic study of *Tamarindus indica* L. organs using GC/MS analysis demonstrated that triterpenoids and steroids are the predominant classes in the bark (TIB) (61.06% and 13.47%, respectively) and that these compounds exhibit significant anti-inflammatory and wound-healing activities. In vitro scratch assay using Human Skin Fibroblast (HSF) cells was used to assess *Tamarindus indica* L. wound-healing activity. It was evaluated by changes in wound width by measuring the average distance between the borders of the scratches. After 24 h, the highest wound healing potential was recorded by TIB *n*-hexane extracts with wound widths equal to 1.09 ± 0.04 [54].

**Table 1:** Phytochemical classes, their associated molecular pathways, and the effects on wound healing.

Phytochemical Class	Main Signaling Pathways/Molecular Targets	Wound-Healing Effects	References
**Tannins**	ROS scavenging; activation of antioxidant enzymes (e.g., SOD, CAT); metal ion chelation	Neutralize reactive oxygen species (ROS); reduce oxidative stress; protect cellular components; accelerate tissue repair;	[45,46,47]
**Alkaloids**	NF-κB pathway inhibition; downregulation of pro-inflammatory cytokines (IL-1β, TNF-α)	Suppress inflammation; reduce cytokine-mediated tissue damage;	[50,51]
**Flavonoids**	NF-κB, MAPK, and COX/LOX inhibition; regulation of arachidonic acid metabolism; antioxidant enzyme activation	Reduce inflammation; protect against oxidative stress; stimulate fibroblast and keratinocyte proliferation and migration;	[47,52,53,54]
**Terpenes**	Modulation of inflammatory mediators, chemokines, and growth factors; enhancement of granulation tissue and epithelialization	Accelerate wound contraction, collagen deposition, and tissue remodeling;	[54,55,56]

## 7. Methods for Studying the Molecular and Cellular Mechanisms of Wound Healing

### 7.1. In Vitro Studies–Effects on Keratinocyte, Fibroblast, and Macrophage Cell Lines

**Figure 8:**
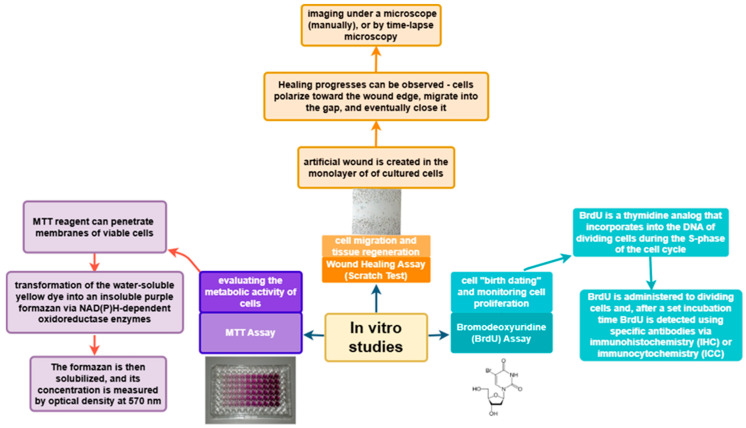
In vitro studies of wound healing.

In vitro studies provide valuable information on the biological activity of plant extracts and compounds on the main cells involved in the skin wound healing process–keratinocytes, fibroblasts, and macrophages. Several tests exist, but the most commonly used methods are MTT assay, BrdU incorporation, wound healing assay (scratch test), and ELISA to assess cell viability, proliferation, migration, and secretion of inflammatory mediators (Figure 8).

#### 7.1.1. MTT Assay–Assessment of Metabolic Activity and Cell Viability

The MTT (3-(4,5-dimethylthiazol-2-yl)-2,5-diphenyltetrazolium bromide) assay is one of the most widely used in vitro colorimetric analyses. It is based on the evaluation of cellular metabolic activity. The MTT reagent can cross both the cell membrane and the inner mitochondrial membrane of viable cells due to its lipophilic nature and positive charge. It is reduced to formazan by metabolically active cells [57]. The conversion of the water-soluble yellow dye to insoluble purple formazan is catalyzed by NAD(P)H-dependent oxidoreductase enzymes. The resulting formazan is then dissolved, and its concentration is determined by measuring optical density at 570 nm [58].

The cytotoxicity of the ethanol extract from the plant material of Glycyrrhiza glabra was evaluated using the MTT assay on the Vero cell line, which is one of the most widely used in vitro mammalian models in scientific research. The results of the MTT assay indicate that even at elevated concentrations, the *G. glabra* extracts do not exhibit toxic effects on the cells [59].

#### 7.1.2. BrdU Assay–Measurement of Cell Proliferation

Bromodeoxyuridine (BrdU) is a thymidine analog that is incorporated into the DNA of dividing cells during the S-phase of the cell cycle. This property allows it to be used for determining cell “birthdating” and monitoring cell proliferation. BrdU is either injected or added to dividing cells, and after a defined period, the tissues are fixed. BrdU incorporation is then detected using specific antibodies through methods such as immunohistochemistry (IHC) or immunocytochemistry (ICC) [60].

#### 7.1.3. Wound Healing Assay (Scratch Test)–Assessment of Cell Migration and Tissue Repair

The wound healing assay, also known as the scratch test, is a widely used method for evaluating cell migration and tissue repair. After injury or scratching of cell monolayers, intercellular junctions are disrupted, leading to a localized increase in the concentration of growth factors and cytokines at the wound site. This biochemical response activates the proliferation and migration of various cell types, such as keratinocytes and fibroblasts, which are key participants in tissue repair processes.

In this in vitro assay, an artificial wound is created in the monolayer by mechanically scratching with a sharp instrument, typically the tip of a pipette. The model can be applied to coverslips or in multi-well culture plates to simulate wound closure in vitro. Healing occurs in stages: cells polarize toward the wound edges, form protrusions, migrate into the gap, and gradually fill it. Progress can be monitored by fixing cell samples at different time points and imaging them manually under a microscope, or by using time-lapse microscopy, which allows real-time visualization of cell dynamics [61,62].

To evaluate the potential of a given extract or compound to promote wound re-epithelialization and ensure quality control, the use of a standard is necessary. Several growth factors and cytokines influence fibroblast motility. Due to its well-characterized role in wound healing, platelet-derived growth factor (PDGF) is often used as a positive control in such experiments. PDGF stimulates fibroblasts to synthesize key components of the extracellular matrix, including fibronectin, collagen, and hyaluronic acid, as well as enzymes such as collagenases, which participate in tissue remodeling [63].

The main limitations of the method include uneven scratches when manually creating the wound and potential disruption of extracellular matrix coatings on the culture surface [64]. Additionally, displaced cells can accumulate at the edges of the artificial gap, complicating data analysis and potentially affecting subsequent proliferation and migration required for wound closure [65,66].

The wound healing assay of two different extracts from *Calendula officinalis* flowers demonstrated that the hexane extract increased cell numbers by 54.76% ± 1.59 at 1 μg/mL and 64.35% ± 1.60 at 10 μg/mL. The ethyl extract showed slightly higher results, increasing cell numbers by 60.80% ± 4.36 at 1 μg/mL and 70.53% ± 2.64 at 10 μg/mL. These findings indicate that *Calendula officinalis*, particularly in its ethyl extract form, can stimulate fibroblast proliferation, supporting its traditional use in wound healing [62].

### 7.2. In Vivo Models–Experimental Animal Studies (Mice, Rats, Rabbits)

In vivo models represent the most effective alternative for studying wound healing processes. In comparison to in vitro models, they provide a number of advantages related to the possibility of monitoring the complex pathophysiological mechanisms of tissue regeneration in real time and in conditions that more accurately reflect the natural biological environment of the organism. Due to their high biological applicability, in vivo models are an indispensable stage in the preclinical evaluation of new therapeutic agents [67].

The anatomical and physiological characteristics of the skin in rodents differ from those in humans, which should be taken into account when interpreting the results of experimental in vivo models. Rodent skin is characterized by a thinner epidermis, poorer adhesion to underlying tissues, and dense hair growth, which is considered to accelerate wound healing processes [68]. There is also a subcutaneous muscle layer, known as the *panniculus carnosus*, which facilitates wound contraction and leads to faster mechanical closure of skin defects. Additionally, rodents have a more pronounced immune reactivity compared to humans [69]. Despite the above-described differences, rodents remain a preferred model in wound healing studies due to their wide availability, low cost, and small size. These characteristics make them highly suitable for conducting experiments with a large number of animals, which increases the statistical reliability of the results and allows for a preliminary assessment of the efficacy and safety of new therapeutic agents before moving on to human clinical trials [70].

Histological preparations taken from a rat excision wound model treated with *Pupalia lappacea* (L.) *Juss* extracts showed significantly increased collagen formation, re-epithelialization, granulation tissue formation, and angiogenesis compared to untreated control wounds [71]. In an excision wound model, the methanolic extract of *Myrianthus arboreus* leaves showed significantly (*p* < 0.05) accelerated wound closure, and histological examinations of the treated tissues revealed high fibrosis and collagenation compared to untreated rat wounds [72].

Experimental studies have shown that the ethanolic extract of the leaves of *Hibiscus rosa-sinensis* L., administered at a dose of 120 mg/kg daily, leads to a significant reduction in wound area by 86%, compared to a 75% reduction in the control group in rats. In animals treated with the extract, statistically significant epithelialization (*p* < 0.002) was observed, as well as increased skin tear strength compared to controls (*p* < 0.002). In addition, a significant increase in the wet and dry weight of granulation tissue was reported, as well as in the hydroxyproline content, which is an indicator of increased collagen synthesis [73].

In in vivo models with BALB (Bagg Albino Laboratory-Bred) mice, it has been found that extracts of *Calendula officinalis* flowers support the wound healing process by favoring the formation of granulation tissue. This effect is accompanied by the regulation of the expression of key markers associated with healing—connective tissue growth factor (CTGF) and α-smooth muscle actin (α-SMA) [74,75].

Methanolic extract of *Camellia sinensis* stimulates fibroblast proliferation and collagen synthesis. Data from in vivo studies in rats show that the plant significantly promotes wound healing by increasing angiogenesis and has a positive effect on wound healing in a diabetic mouse model, which highlights its potential in impaired regeneration processes [75,76,77].

Although numerous in vitro and in vivo studies have demonstrated the beneficial effects of medicinal plant extracts and their bioactive compounds on wound healing, the heterogeneity of experimental models and treatment conditions makes direct comparison challenging. Differences in animal species, wound induction methods, and extract standardization limit translational relevance to human applications. Moreover, while the plants that are mentioned have strong experimental support, evidence for other phytochemicals remains preliminary and largely confined to preclinical investigations. Therefore, future studies should prioritize standardized methodologies and clinical validation to better establish the therapeutic potential and safety profiles of these natural compounds (Table 2).

**Table 2:** Medicinal plants with demonstrated wound-healing activity, experimental models, and observed effects.

Species (Family)	Method	Effect	Reference
*Ficus trijuja*	In vivo: Topical application of lipid nanocapsules (LNCs) on rat skin	significantly enhanced wound healing while down-regulating TNF-α mRNA and IL-1β protein expression, indicating strong anti-inflammatory activity increase in VEGF gene expression	[78]
*Glycyrrhiza glabra and Sophora japonica*	In vivo: Wound closure assay—topical application of ointment on rat skin	significantly improved wound contraction increased the GSH levels	[54]
*Hamamelis virginiana*	In vitro: HaCaT Cells (human keratinocytes) MTT assay for cell viability	effectively inhibited NF-κB transcriptional activity inhibits IL-4-induced responses in keratinocytes no cytotoxic effects on HaCaT cells	[48]
*Syzygium cumini*	In vivo: Wound closure assay—topical application of *Syzygium cumini*; loaded electrospun nanofibers	remarkable wound healing, with full closure occurring between days 12 and 14	[79]
*Tamarindus indica*	In vitro: Scratch Wound Assay using Human Skin Fibroblast cells (HSF)	both bark and leaves n-hexane extracts exhibited almost complete cell migration after 48 h of observation	[80]
*Citrus x macrocarpa leaves*	In vitro: MTT assay method Fibroblast Cell Migration with Scratch Assay Method	leaves significantly promoted fibroblast proliferation wound closure percentage of 77.54%.	[81]
*Matricaria chamomilla*	In vivo: Chamomile compresses in perianal skin lesions in colostomy patients	wound healing was observed in 100% of the chamomile group compared to the 1% hydrocortisone ointment group (76%)	[82]
*Agrimonia eupatoria* L.	In vitro: HaCaT cell line (human keratinocytes) In vivo: Wounds in male Sprague–Dawley rats	increased cell migration, proliferation and collagen IV synthesis; increased phosphorylation of ERK1/2, JNK, MAPK p38 and Akt significantly higher wound tensile strength (TS) compared to the two control groups; significantly increased contraction rates compared to the control groups	[83]
*Carum carvi*	In vivo: Wounds in male Sprague–Dawley rats	More complete re-epithelialization, greater granulation tissue maturity, increased connective tissue density, and higher collagen fiber content; an increase in collagen and elastin fibers	[84]
*Centella asiatica*	In vitro: HaCaT cell line (human keratinocytes)	stimulated cell migration, accelerating wound closure compared to the untreated control	[85]
*Glycyrrihza glabra*	In vivo: Wounds in male Wister rats	SOD, GPx activities, and GSH content increased. MDA levels (oxidative stress marker) decreased Complete re-epithelialization in treated groups. Collagen synthesis significantly increased	[86]
*Cynara humilis*	In vivo: Burn wounds in male Wister rats—topical use of *C. humilis* ointment	Accelerating epithelialization and collagen deposition Reducing inflammatory cell infiltration production of a modest amount of extracellular matrix and the development of new blood vessels and hair follicles	[87]
*Pupalia lappacea* (L.)	In vivo: Wounds in male Sprague–Dawley rats	Increased collagen formation, re-epithelialization, granulation tissue formation, and angiogenesis compared to untreated control wounds	[71]
*Myrianthus arboreus*	In vivo: Wounds in male Sprague–Dawley rats	Significantly accelerated wound closure Increased proliferation of fibroblasts and collagen formation in the treated wound tissues	[72]
*Camellia sinensis*	In vivo: Models with BALB (Bagg Albino Laboratory-Bred) mice	Stimulates fibroblast proliferation and collagen synthesis Promotes wound healing by increasing angiogenesis and has a positive effect on wound healing in a diabetic mouse model	[75,76,77]
*Hibiscus rosa-sinensis* L.	In vivo: Wounds in male rats	Significant reduction in wound area Increased epithelialization and skin tear strength Increase in the wet and dry weight of granulation tissue and hydroxyproline content	[73]

## 8. Conclusions

The molecular mechanisms of skin wound healing involve complex interactions between keratinocytes, fibroblasts, macrophages, growth factors, cytokines, and signaling pathways such as NF-κB, MAPK, and STAT3. Medicinal plants and their bioactive compounds, including flavonoids, alkaloids, tannins, and other phytoconstituents, can modulate these molecular processes through anti-inflammatory, antioxidant, and profibrotic effects. This indicates that plant extracts represent promising tools to support skin reparative mechanisms by simultaneously stimulating proliferation and migration of key cells, reducing oxidative stress, and accelerating wound healing. However, further research, including clinical trials, is needed to optimize their doses, types of application, and mechanistic effects in human tissue.

## 9. Future Directions

Despite growing evidence for the therapeutic effects of medicinal plants in wound healing, translation into clinical practice remains limited. Future research should prioritize the development of advanced drug delivery systems capable of improving stability, bioavailability, and targeted action of plant compounds. Localized delivery strategies allow sustained release of bioactive compounds at the wound site, minimize systemic exposure, and can be engineered to respond to the dynamic microenvironment of healing tissue. Integrating plant-derived bioactive compounds with modern biomaterials offers a promising approach for next-generation wound therapeutics. Further preclinical and clinical studies are needed to optimize local delivery systems, ensuring safety, reproducibility, and efficacy.

## Data Availability

Not applicable.

## References

[1] Rodrigues M., Kosaric N., Bonham C.A., Gurtner G.C. (2019). Wound Healing: A Cellular Perspective. Physiol. Rev..

[2] Jiao Q., Zhi L., You B., Wang G., Wu N., Jia Y. (2024). Skin Homeostasis: Mechanism and Influencing Factors. J. Cosmet. Dermatol..

[3] Olutoye O.O., Eriksson E., Menchaca A.D., Kirsner R.S., Tanaka R., Schultz G., Weir D., Wagner T.L., Fabia R.B., Naik-Mathuria B. (2024). Management of acute wounds—Expert panel consensus statement. Adv. Wound Care.

[4] Lordani T.V.A., de Lara C.E., Ferreira F.B.P., de Souza Terron Monich M., Mesquita da Silva C., Felicetti Lordani C.R., Lonardoni M.V.C. (2018). Therapeutic Effects of Medicinal Plants on Cutaneous Wound Healing in Humans: A Systematic Review. Mediators Inflamm..

[5] Cedillo-Cortezano M., Martinez-Cuevas L.R., López J.A.M., Barrera López I.L., Escutia-Perez S., Petricevich V.L. (2024). Use of Medicinal Plants in the Process of Wound Healing: A Literature Review. Pharmaceuticals.

[6] Zhu X., Olsson M.M., Bajpai R., Järbrink K., Tang W.E., Car J. (2022). Health-Related Quality of Life and Chronic Wound Characteristics among Patients with Chronic Wounds Treated in Primary Care: A Cross-Sectional Study in Singapore. Int. Wound J..

[7] Rustamova T., Plate S. (2025). From Chronic Leg Ulcer to Squamous Cell Carcinoma: A Case Report. Eur. J. Med. Health Sci..

[8] Rumbaut R.E., Thiagarajan P. (2010). Platelet–Vessel Wall Interactions in Hemostasis and Thrombosis.

[9] Godo S., Shimokawa H. (2017). Endothelial Functions. Arterioscler. Thromb. Vasc. Biol..

[10] Eming S.A., Martin P., Tomic-Canic M. (2014). Wound Repair and Regeneration: Mechanisms, Signaling, and Translation. Sci. Transl. Med..

[11] MacLeod A.S., Mansbridge J.N. (2016). The Innate Immune System in Acute and Chronic Wounds. Adv. Wound Care.

[12] Ellis S., Lin E.J., Tartar D. (2018). Immunology of Wound Healing. Curr. Dermatol. Rep..

[13] Hart J. (2002). Inflammation 1: Its Role in the Healing of Acute Wounds. J. Wound Care.

[14] Krzyszczyk P., Schloss R., Palmer A., Berthiaume F. (2018). The Role of Macrophages in Acute and Chronic Wound Healing and Interventions to Promote Pro-Wound Healing Phenotypes. Front. Physiol..

[15] Madej M.P., Töpfer E., Boraschi D., Italiani P. (2017). Different Regulation of Interleukin-1 Production and Activity in Monocytes and Macrophages: Innate Memory as an Endogenous Mechanism of IL-1 Inhibition. Front. Pharmacol..

[16] Werner S., Krieg T., Smola H. (2007). Keratinocyte–Fibroblast Interactions in Wound Healing. J. Investig. Dermatol..

[17] Peng Y., Wu S., Tang Q., Li S., Peng C. (2019). KGF-1 Accelerates Wound Contraction through the TGF-β1/Smad Signaling Pathway in a Double-Paracrine Manner. J. Biol. Chem..

[18] Velnar T., Bailey T., Smrkolj V. (2009). The Wound Healing Process: An Overview of the Cellular and Molecular Mechanisms. J. Int. Med. Res..

[19] Lau K., Paus R., Tiede S., Day P., Bayat A. (2009). Exploring the Role of Stem Cells in Cutaneous Wound Healing. Exp. Dermatol..

[20] Fantin A., Vieira J.M., Gestri G., Denti L., Schwarz Q., Prykhozhij S., Ruhrberg C. (2010). Tissue Macrophages Act as Cellular Chaperones for Vascular Anastomosis Downstream of VEGF-Mediated Endothelial Tip Cell Induction. Blood.

[21] Brancato S.K., Albina J.E. (2011). Wound Macrophages as Key Regulators of Repair: Origin, Phenotype, and Function. Am. J. Pathol..

[22] Lucas T., Waisman A., Ranjan R., Roes J., Krieg T., Müller W., Eming S.A. (2010). Differential Roles of Macrophages in Diverse Phases of Skin Repair. J. Immunol..

[23] Yannas I.V., Tzeranis D.S., So P.T. (2017). Regeneration of Injured Skin and Peripheral Nerves Requires Control of Wound Contraction, Not Scar Formation. Wound Repair Regen..

[24] Aggarwal B.B., Shishodia S., Sandur S.K., Pandey M.K., Sethi G. (2006). Inflammation and Cancer: How Hot Is the Link?. Biochem. Pharmacol..

[25] Jang D.I., Lee A.H., Shin H.Y., Song H.R., Park J.H., Kang T.B., Yang S.H. (2021). The Role of Tumor Necrosis Factor Alpha (TNF-α) in Autoimmune Disease and Current TNF-α Inhibitors in Therapeutics. Int. J. Mol. Sci..

[26] Balkwill F., Mantovani A. (2010). Cancer and Inflammation: Implications for Pharmacology and Therapeutics. Clin. Pharmacol. Ther..

[27] Heinrich P.C., Behrmann I., Haan S., Hermanns H.M., Müller-Newen G., Schaper F. (2003). Principles of Interleukin (IL)-6-Type Cytokine Signalling and Its Regulation. Biochem. J..

[28] Tasneem S., Liu B., Li B., Choudhary M.I., Wang W. (2019). Molecular Pharmacology of Inflammation: Medicinal Plants as Anti-Inflammatory Agents. Pharmacol. Res..

[29] Yang L., Pang Y., Moses H.L. (2010). TGF-β and Immune Cells: An Important Regulatory Axis in the Tumor Microenvironment and Progression. Trends Immunol..

[30] Massagué J., Wotton D. (2000). Transcriptional Control by the TGF-β/Smad Signaling System. EMBO J..

[31] Bivolarska A. (2025). Medical Biochemistry–Part II.

[32] Napetschnig J., Wu H. (2013). Molecular Basis of NF-κB Signaling. Annu. Rev. Biophys..

[33] Zhang T., Ma C., Zhang Z., Zhang H., Hu H. (2021). NF-κB Signaling in Inflammation and Cancer. MedComm.

[34] Chauhan A., Islam A.U., Prakash H., Singh S. (2022). Phytochemicals Targeting NF-κB Signaling: Potential Anti-Cancer Interventions. J. Pharm. Anal..

[35] Boyle D.L., Soma K., Hodge J., Kavanaugh A., Mandel D., Mease P., Firestein G.S. (2015). The JAK Inhibitor Tofacitinib Suppresses Synovial JAK1-STAT Signalling in Rheumatoid Arthritis. Ann. Rheum. Dis..

[36] Yin Q., Wang L., Yu H., Chen D., Zhu W., Sun C. (2021). Pharmacological Effects of Polyphenol Phytochemicals on the JAK-STAT Signaling Pathway. Front. Pharmacol..

[37] Kaminska B. (2005). MAPK Signalling Pathways as Molecular Targets for Anti-Inflammatory Therapy—From Molecular Mechanisms to Therapeutic Benefits. Biochim. Biophys. Acta.

[38] Thiel M.J., Schaefer C.J., Lesch M.E., Mobley J.L., Dudley D.T., Tecle H., Flory C.M. (2007). Central Role of the MEK/ERK MAP Kinase Pathway in a Mouse Model of Rheumatoid Arthritis: Potential Proinflammatory Mechanisms. Arthritis Rheum..

[39] Shi A., Liu L., Li S., Qi B. (2024). Natural Products Targeting the MAPK-Signaling Pathway in Cancer: Overview. J. Cancer Res. Clin. Oncol..

[40] Fialho L., Cunha-E-Silva J.A., Santa-Maria A.F., Madureira F.A., Iglesias A.C. (2018). Comparative Study of Systemic Early Postoperative Inflammatory Response among Elderly and Non-Elderly Patients Undergoing Laparoscopic Cholecystectomy. Rev. Col. Bras. Cir..

[41] Lukova P., Apostolova E., Baldzhieva A., Murdjeva M., Kokova V. (2023). Fucoidan from Ericaria crinita Alleviates Inflammation in Rat Paw Edema, Downregulates Pro-Inflammatory Cytokine Levels, and Shows Antioxidant Activity. Biomedicines.

[42] Lukova P., Kokova V., Baldzhieva A., Murdjeva M., Katsarov P., Delattre C., Apostolova E. (2024). Alginate from Ericaria crinita Possesses Antioxidant Activity and Attenuates Systemic Inflammation via Downregulation of Pro-Inflammatory Cytokines. Mar. Drugs.

[43] Stavrakeva K., Metodieva K., Benina M., Bivolarska A., Dimov I., Choneva M., Kokova V., Alseekh S., Ivanova V., Vatov E. (2024). Metabolic Composition of Methanolic Extract of the Balkan Endemic Species *Micromeria frivaldszkyana* (*Degen*) Velen and Its Anti-Inflammatory Effect on Male Wistar Rats. Int. J. Mol. Sci..

[44] Nunes C.d.R., Barreto Arantes M., Menezes de Faria Pereira S., Leandro da Cruz L., de Souza Passos M., Pereira de Moraes L., Vieira I.J.C., Barros de Oliveira D. (2020). Plants as Sources of Anti-Inflammatory Agents. Molecules.

[45] Xie L., Roto A.V., Bolling B.W. (2012). Characterization of Ellagitannins, Gallotannins, and Bound Proanthocyanidins from California Almond (*Prunus dulcis*) Varieties. J. Agric. Food Chem..

[46] Schieber M., Chandel N.S. (2014). ROS Function in Redox Signaling and Oxidative Stress. Curr. Biol..

[47] Shen N., Wang T., Gan Q., Liu S., Wang L., Jin B. (2022). Plant Flavonoids: Classification, Distribution, Biosynthesis, and Antioxidant Activity. Food Chem..

[48] Piazza S., Martinelli G., Magnavacca A., Fumagalli M., Pozzoli C., Terno M., Sangio-vanni E. (2022). Unveiling the ability of witch hazel (*Hamamelis virginiana* L.) bark extract to impair keratinocyte inflammatory cascade typical of atopic eczema. Int. J. Mol. Sci..

[49] Mondal A., Gandhi A., Fimognari C., Atanasov A.G., Bishayee A. (2019). Alkaloids for Cancer Prevention and Therapy: Current Progress and Future Perspectives. Eur. J. Pharmacol..

[50] Batool S., Asim L., Zhang J., Qureshi F.R., Saleem R.S.Z. (2025). Anti-Inflammatory Alkaloids Targeting IL-1 against Respiratory Viral Infections: A Special Insight into Drug Development against SARS-CoV-2. Mini-Rev. Org. Chem..

[51] Han X., Ma T., Wang Q., Jin C., Han Y., Liu G., Li H. (2023). The mechanism of oxymatrine on atopic dermatitis in mice based on SOCS1/JAK-STAT3 pathway. Front. Pharmacol..

[52] Bassiouni W., Daabees T., Louedec L., Norel X., Senbel A.M. (2019). Evaluation of Some Prostaglandins Modulators on Rat Corpus Cavernosum In-Vitro: Is Relaxation Negatively Affected by COX-Inhibitors?. Biomed. Pharmacother..

[53] Zuo A.R., Dong H.H., Yu Y.Y., Shu Q.L., Zheng L.X., Yu X.Y., Cao S.W. (2018). The Antityrosinase and Antioxidant Activities of Flavonoids Dominated by the Number and Location of Phenolic Hydroxyl Groups. Chin. Med..

[54] Aly S.H., Elissawy A.M., Mahmoud A.M.A., El-Tokhy F.S., Mageed S.S.A., Almahli H., Al-Rashood S.T., Binjubair F.A., Hassab M.A.E., Eldehna W.M. (2023). Synergistic Effect of Sophora japonica and Glycyrrhiza glabra Flavonoid-Rich Fractions on Wound Healing: In Vivo and Molecular Docking Studies. Molecules.

[55] Pereira Beserra F., Xue M., Maia G.L.d.A., Leite Rozza A., Helena Pellizzon C., Jackson C.J. (2018). Lupeol, a Pentacyclic Triterpene, Promotes Migration, Wound Closure, and Contractile Effect In Vitro: Possible Involvement of PI3K/Akt and p38/ERK/MAPK Pathways. Molecules.

[56] Pereira Beserra F., Sérgio Gushiken L.F., Vieira A.J., Augusto Bérgamo D., Luísa Bérgamo P., Oliveira de Souza M., Alberto Hussni C., Kiomi Takahira R., Henrique Nóbrega R., Monteiro Martinez E.R. (2020). From Inflammation to Cutaneous Repair: Topical Application of Lupeol Improves Skin Wound Healing in Rats by Modulating the Cytokine Levels, NF-κB, Ki-67, Growth Factor Expression, and Distribution of Collagen Fibers. Int. J. Mol. Sci..

[57] Ghasemi M., Turnbull T., Sebastian S., Kempson I. (2021). The MTT Assay: Utility, Limitations, Pitfalls, and Interpretation in Bulk and Single-Cell Analysis. Int. J. Mol. Sci..

[58] Kumar P., Nagarajan A., Uchil P.D. (2018). Analysis of Cell Viability by the MTT Assay. Cold Spring Harb. Protoc..

[59] Roy I., Magesh K.T., Sathyakumar M., Sivachandran A., Purushothaman D., Aravindhan R. (2023). Evaluation of Wound Healing Property of the Ethanolic Extract of Glycyrrhiza glabra on Vero Cell Lines Using In Vitro Scratch Assay Test. J. Pharm. Bioallied Sci..

[60] Taupin P. (2007). BrdU Immunohistochemistry for Studying Adult Neurogenesis: Paradigms, Pitfalls, Limitations, and Validation. Brain Res. Rev..

[61] Liang C.C., Park A.Y., Guan J.L. (2007). In Vitro Scratch Assay: A Convenient and Inexpensive Method for Analysis of Cell Migration In Vitro. Nat. Protoc..

[62] Fronza M., Heinzmann B., Hamburger M., Laufer S., Merfort I. (2009). Determination of the Wound Healing Effect of Calendula Extracts Using the Scratch Assay with 3T3 Fibroblasts. J. Ethnopharmacol..

[63] Li W., Fan J., Chen M., Guan S., Sawcer D., Bokoch G.M., Woodley D.T. (2004). Mechanism of Human Dermal Fibroblast Migration Driven by Type I Collagen and Platelet-Derived Growth Factor-BB. Mol. Biol. Cell.

[64] Goetsch K.P., Niesler C.U. (2011). Optimization of the Scratch Assay for In Vitro Skeletal Muscle Wound Healing Analysis. Anal. Biochem..

[65] Ashby W.J., Zijlstra A. (2012). Established and Novel Methods of Interrogating Two-Dimensional Cell Migration. Integr. Biol..

[66] Stamm A., Reimers K., Strauß S., Vogt P., Scheper T., Pepelanova I. (2016). In Vitro Wound Healing Assays–State of the Art. BioNanoMaterials.

[67] Stephens P., Caley M., Peake M. (2013). Alternatives for Animal Wound Model Systems. Wound Regeneration and Repair: Methods and Protocols.

[68] Dorsett-Martin W.A., Wysocki A.B. (2008). Rat Models of Skin Wound Healing. Sourcebook of Models for Biomedical Research.

[69] Seaton M., Hocking A., Gibran N.S. (2015). Porcine Models of Cutaneous Wound Healing. ILAR J..

[70] Trøstrup H., Thomsen K., Calum H., Høiby N., Moser C. (2016). Animal Models of Chronic Wound Care: The Application of Biofilms in Clinical Research. Chronic Wound Care Manag. Res..

[71] Apenteng J.A., Agyare C., Adu F., Ayande P.G., Boakye Y.D. (2014). Evaluation of Wound Healing Potential of Different Leaf Extracts of Pupalia lappacea. Afr. J. Pharm. Pharmacol..

[72] Agyare C., Ansah A.O., Ossei P.P.S., Apenteng J.A., Boakye Y.D. (2014). Wound Healing and Anti-Infective Properties of Myrianthus arboreus and Alchornea cordifolia. Med. Chem..

[73] Shivananda Nayak B., Sivachandra Raju S., Orette F.A., Chalapathi Rao A.V. (2007). Effects of *Hibiscus rosa sinensis* L. (*Malvaceae*) on Wound Healing Activity: A Preclinical Study in a Sprague Dawley Rat. Int. J. Low. Extrem. Wounds.

[74] Dinda M., Mazumdar S., Das S., Ganguly D., Dasgupta U.B., Dutta A., Karmakar P. (2016). The Water Fraction of Calendula officinalis Hydroethanol Extract Stimulates In Vitro and In Vivo Proliferation of Dermal Fibroblasts in Wound Healing. Phytother. Res..

[75] Kim H., Kawazoe T., Han D.W., Matsumara K., Suzuki S., Tsutsumi S., Hyon S.H. (2008). Enhanced Wound Healing by an Epigallocatechin Gallate-Incorporated Collagen Sponge in Diabetic Mice. Wound Repair Regen..

[76] Asadi S.Y., Parsaei P., Karimi M., Ezzati S., Zamiri A., Mohammadizadeh F., Rafieian-Kopaei M. (2013). Effect of Green Tea (*Camellia sinensis)* Extract on Healing Process of Surgical Wounds in Rat. Int. J. Surg..

[77] Shedoeva A., Leavesley D., Upton Z., Fan C. (2019). Wound Healing and the Use of Medicinal Plants. Evid.-Based Complement. Altern. Med..

[78] Hashad I.M., Aly S.H., Saleh D.O., Abo El-Nasr N.M.E., Shabana M.E., El-Tokhy F.S., El-Nashar H.A.S., Abdelmohsen U.R., Mostafa N.M., Mostafa A.M. (2025). Mechanistic Wound Healing of Ficus trijuja Leaf Extract and Its Lipid Nanocapsule Supported by Metabolomic Profiling and In Vivo Studies. Int. J. Mol. Sci..

[79] Abdelazim E.B., Abed T., Goher S.S., Alya S.H., El-Nashar H.A., El-Moslamy S.H., Kamoun E.A. (2024). In Vitro and In Vivo Studies of *Syzygium cumini*-Loaded Electrospun PLGA/PMMA/Collagen Nanofibers for Accelerating Topical Wound Healing. RSC Adv..

[80] Aly S.H., El-Hassab M.A., Elhady S.S., Gad H.A. (2023). Comparative Metabolic Study of *Tamarindus indica* L.’s Various Organs Based on GC/MS Analysis, In Silico and In Vitro Anti-Inflammatory and Wound Healing Activities. Plants.

[81] Husni E., Hamidi D., Pavvellin D., Hidayah H., Syafri S. (2024). Metabolite profiling, antioxidant, and in vitro wound healing activities of Citrus medica L. and Citrus × microcarpa Bunge peels and leaves essential oils. Prospect. Pharm. Sci..

[82] Melnyk N., Nyczka A., Piwowarski J.P., Granica S. (2024). Traditional Use of Chamomile Flowers (*Matricariae flos*) in Inflammatory-Associated Skin Disorders. Prospect. Pharm. Sci..

[83] Vasilenko T., Kováč I., Slezák M., Ďurkáč J., Peržel’ová V., Čoma M., Kaňuchová M., Urban L., Szabo P., Dvořánková B. (2022). *Agrimonia eupatoria* L. Aqueous Extract Improves Skin Wound Healing: An In Vitro Study in Fibroblasts and Keratinocytes and In Vivo Study in Rats. In Vivo.

[84] Salari Rafsanjani M., Tabatabaei Naeini A., Meimandi-Parizi A., Nowzari F., Mujtaba Wani M., Iraji A. (2022). Wound Healing Effect of *Carum carvi* L. on the Incised Skin Wound in Male Rats: Histopathology, Total Protein and Biomechanical Evaluations. Vet. Med. Sci..

[85] Nizioł-Łukaszewska Z., Zagórska-Dziok M., Ziemlewska A., Bujak T. (2020). Comparison of the Antiaging and Protective Properties of Plants from the Apiaceae Family. Oxidative Med. Cell. Longev..

[86] Assar D.H., Elhabashi N., Mokhbatly A.A.A., Ragab A.E., Elbialy Z.I., Rizk S.A., Atiba A. (2021). Wound healing potential of licorice extract in rat model: Antioxidants, histopathological, immunohistochemical and gene expression evidences. Biomed. Pharmacother..

[87] Salhi N., El Guourrami O., Rouas L., Moussaid S., Moutawalli A., Benkhouili F.Z., Cherrah Y. (2023). Evaluation of the wound healing potential of *Cynara humilis* extracts in the treatment of skin burns. Evid.-Based Complement. Altern. Med..

